# Automatic detection of epilepsy from EEGs using a temporal convolutional network with a self-attention layer

**DOI:** 10.1186/s12938-024-01244-w

**Published:** 2024-06-01

**Authors:** Leen Huang, Keying Zhou, Siyang Chen, Yanzhao Chen, Jinxin Zhang

**Affiliations:** 1 Department of Medical Statistics, School of Public Health, Sun Yat-sen University, Guangzhou, 510080 Guangdong China; 2 Department of Pediatrics, Shenzhen People’s Hospital, Shenzhen, 518020 Guangdong China; 3 Department of Pediatrics, Second Clinical Medical College of Jinan University, Shenzhen, 518020 Guangdong China; 4 Department of Pediatrics, First Affiliated Hospital of Southern University of Science and Technology, Shenzhen, 518020 Guangdong China

**Keywords:** Convolutional neural network, EEG, Epileptic seizure, Pediatric epilepsy, Attention mechanism

## Abstract

**Background:**

Over 60% of epilepsy patients globally are children, whose early diagnosis and treatment are critical for their development and can substantially reduce the disease’s burden on both families and society. Numerous algorithms for automated epilepsy detection from EEGs have been proposed. Yet, the occurrence of epileptic seizures during an EEG exam cannot always be guaranteed in clinical practice. Models that exclusively use seizure EEGs for detection risk artificially enhanced performance metrics. Therefore, there is a pressing need for a universally applicable model that can perform automatic epilepsy detection in a variety of complex real-world scenarios.

**Method:**

To address this problem, we have devised a novel technique employing a temporal convolutional neural network with self-attention (TCN-SA). Our model comprises two primary components: a TCN for extracting time-variant features from EEG signals, followed by a self-attention (SA) layer that assigns importance to these features. By focusing on key features, our model achieves heightened classification accuracy for epilepsy detection.

**Results:**

The efficacy of our model was validated on a pediatric epilepsy dataset we collected and on the Bonn dataset, attaining accuracies of 95.50% on our dataset, and 97.37% (A v. E), and 93.50% (B vs E), respectively. When compared with other deep learning architectures (temporal convolutional neural network, self-attention network, and standardized convolutional neural network) using the same datasets, our TCN-SA model demonstrated superior performance in the automated detection of epilepsy.

**Conclusion:**

The proven effectiveness of the TCN-SA approach substantiates its potential as a valuable tool for the automated detection of epilepsy, offering significant benefits in diverse and complex real-world clinical settings.

## Background

Epilepsy (EP), a prevalent chronic condition of the nervous system [1], is characterized by irregular neuronal activity and transient cerebral dysfunction resulting from hyper-synchronous discharges. Around 65 million [2] people worldwide suffer from EP, the majority of whom live in low- and middle-income countries. The prevalence of active EP is 6.38%, and the annual incidence is 614.4/10^6^ population [3]. Although the majority of EP patients have a good prognosis and can live ordinary lives, about 35% of them develop refractory EP due to ineffective drug treatments [4]. Childhood EP incidence is notably high [4], with youths representing over 60% of the patient demographics [5, 6]. Moreover, severe EP may hinder a child’s growth and cognitive development, intensifying the familial and social impacts of the disease [7]. Thus, early detection of EP in children is imperative in expediting treatment and minimizing the development of refractory EP.

EEG waveforms indicative of EP are traditionally diagnosed visually by physicians, a method subject to intra-observer variability, thereby diminishing accuracy. As EEGs are multi-channel one-dimensional sequences, it is inefficient to rely on physicians to mark abnormal EEG segments. Given that classification and prediction tasks based on EEG signals are popular multivariable time-series tasks, the automatic identification of EP from EEG signals has long been a research topic of interest to clinical physicians. The advent of machine learning in computing has enhanced the automated analysis of EP [8, 9], demonstrating promising classification capabilities across time [10–12], frequency [13, 14], and time–frequency domains [15], as well as measures of complexity and synchrony [16–22].

Methods based on machine learning have performed well but have the following limitations. First, feature extraction is highly operator-dependent, introducing subjectivity [23]. Second, EEGs inherently feature low signal-to-noise ratios and are susceptible to artifacts from both environmental noise and patient movement, complicating the analysis [23]. Third, the normal EEGs of neurologically asymptomatic individuals can exhibit minor variations [24]. Therefore, automatic identification models based on EEG signals should have high stability that can accommodate person-to-person and temporal differences.

The rapid increase in computing power has made deep learning the fastest-developing branch of machine learning. Its good generalisability, high accuracy, and high stability, coupled with its network architecture, make the technique suitable for the discovery of high-dimensional features and potential associations [23]. Three types of deep learning are currently used for the automatic identification of EEG signals. The first is a convolutional neural network (CNN) [25–28]. Acharya et al. [26] proposed a 13-layer deep CNN for identifying targets from normal, interictal, and seizure EEGs. A system was proposed by Thomas et al. [29] to classify EP based on the interictal EEG, which consists of a Convolutional Neural Network (CNN)-based IED detector, a Template Matching-based IED detector, and a spectral feature-based classifier, and which yielded a mean Leave-One-Institution-Out cross-validation area under curve (AUC) of 0.826 on datasets from six centers. The second is a recurrent neural network (RNN) [30–33]. Li et al. [30] developed a fully convolutional long-term memory, which showed 97.62% sensitivity with the Freiburg hospital database and 94.07% sensitivity with the Children’s Hospital Boston-Massachusetts Institute of Technology scalp EEG database. The third combines two or more neural networks to obtain the main architecture and constructs a deep learning network; for instance, any neural network [34]. Besides, some recent innovations in deep learning have been utilized for the automatic detection task of EP, such as the attention mechanism [35]. Currently, little research has been done using attention mechanisms for EP detection using EEG signals [36, 37].

Although CNNs and RNNs have shown good performance in the automatic detection of EP, they have inherent limitations. In the face of sequential tasks, CNNs, which simulate human visual perception in the receptive field, must limit their receptive fields as one cannot predict current and historical information based on future information. Therefore, when employing EEG sequence data, it is imperative to constrain the receptive field’s orientation appropriately. Parameters such as hyper-parameters can drastically affect the performance of CNNs. RNNs are the preferred network architectures for multivariate time-series-related tasks. However, RNNs may also face the problem of gradients exploding or vanishing during gradient descent when applied to long sequences. For the automatic detection task of EP, RNNs still struggle to capture a sufficient abnormal activity information of temporal context under sequences of finite length for highly accurate predictions of EP. Presently, this problem only can only be lessened by the improvement of RNNs like long short-term memory (LSTM) and gated recurrent unit (GRU), and cannot be solved. Besides, the working mode of RNNs cannot support parallel computing, and RNNs require a long computing time, because they compute the output of each moment by depending on that of the previous moment. If their face difficulties when analyzing sequences with long-term associations [38–40], further limiting it development for sequence tasks.

Combining the advantages of CNNs with those of RNNs, Lea et al. [41, 42] proposed a new convolutional method and constructed temporal convolutional neural networks (TCNs). These TCNs captured the long-term associations of sequences with variable-size receptive fields by flexibly changing the dilated values [43, 44]. Models utilizing residual connections not only ensure that the input and output lengths are the same but also address the problem of gradients exploding or vanishing, which is faced by RNNs. Unlike CNNs, TCNs support parallel computing, can handle different types of time-series tasks, and perform better than traditional sequential modeling networks such as RNNs [45–49]. For the automatic detection of EP, Zhang et al. [50] utilized a TCN to classify the Bonn dataset [51] and achieved excellent performance.

However, TCN has some insufficiencies for the automatic detection of EP, especially when the EEG recordings have the period of interictal and seizure at the same time. Interictal and seizure EEG have different patterns. In the real-world EEG examination, the proportion of the interictal period and the ictal period in EP are highly imbalanced. Sometimes, seizures may not manifest during an entire EEG examination. When it extracts features information from EEG sequences with causal and dilated convolution at equal intervals, TCN will learns the interictal and seizure EEG with the same weight, which will lead to inflated efficiency metrics. Therefore, it is not suitable for the task of EP in the real world. Consequently, a more adaptable model suited for the complexity of real-world EP detection is essential—one that can handle various conditions, including recordings capturing solely interictal activity.

This problem can be elegantly addressed by combining TCNs with self-attention (SA) layers. The TCN-SA model was first proposed by Dai et al. [53] for the detection of daily living activities in long-term untrimmed videos. Our study is the first to use a TCN-SA model for the automatic detection of EP in the real world. For the automatic detection task of EP, by learning the interictal abnormal activities and epileptic seizures, our deep learning model extracted their related information as potential features. In the TCN-SA model, the TCN block not only ensures computation at the current moment to circumvent the influence of future information but also captures sufficient abnormal activity information with flexible size in the receptive fields for highly accurate predictions of EP. Additionally, the SA block adapts the learning weights during network training to recognize seizures, reducing computational demands and enhancing accuracy. We evaluated the TCN-SA model’s performance against other deep learning models (TCN, SA and standard CNN models), using cross-validation to confirm its stability and reliability.

The rest of this article is organized as follows. Sect. “Results” introduces the TCN-SA model and related theory. Sect. “Discussion” describes the datasets and experimental settings. The experimental results are listed in Sect. “Conclusions”, where we also describe the utilization of cross-validation to evaluate the stability and reliability of the TCN-SA model. Sect. “Methods” comprises the discussion, and Sect. 6 concludes the study.

## Results

### Our EEG dataset

**Fig. 1:**
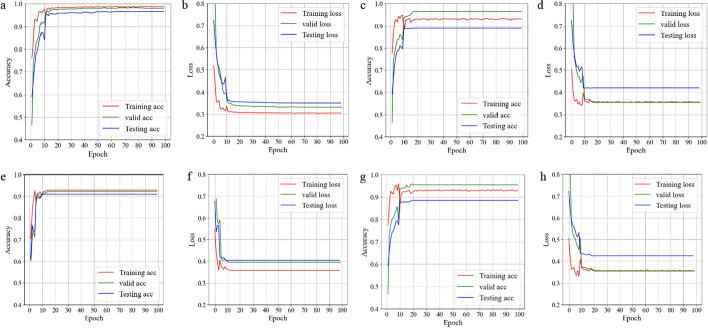
Accuracy and loss of the training, valid, test results of the four models. **a**, **b** TCN-SA model; **c**, **d** TCN model; **e**, **f** CNN model; and **g**, **h** SA model

Our EEG dataset was randomly divided into training and testing sets at a ratio of 7:3. We then used the TCN-SA model to identify patients with EP based on our EEG data. The training accuracy, test accuracy, training loss, and test loss were obtained, as shown in Fig. 1a, b. Additionally, to compare with our model, we implemented three deep learning models on the same dataset: a standard CNN, an SA neural network, and a TCN. The specific training and test results are shown in Fig. 1c–h.

Figure 1 shows that the TCN-SA model had the highest training and testing accuracy and the lowest training and testing loss among the four models. In terms of training and testing, that of TCN-SA model decreased at the fastest speed; it also had a lower final training and test loss than the TCN model. This result indicated that adding the SA layer improved the training and test accuracy of the model.

**Fig. 2:**
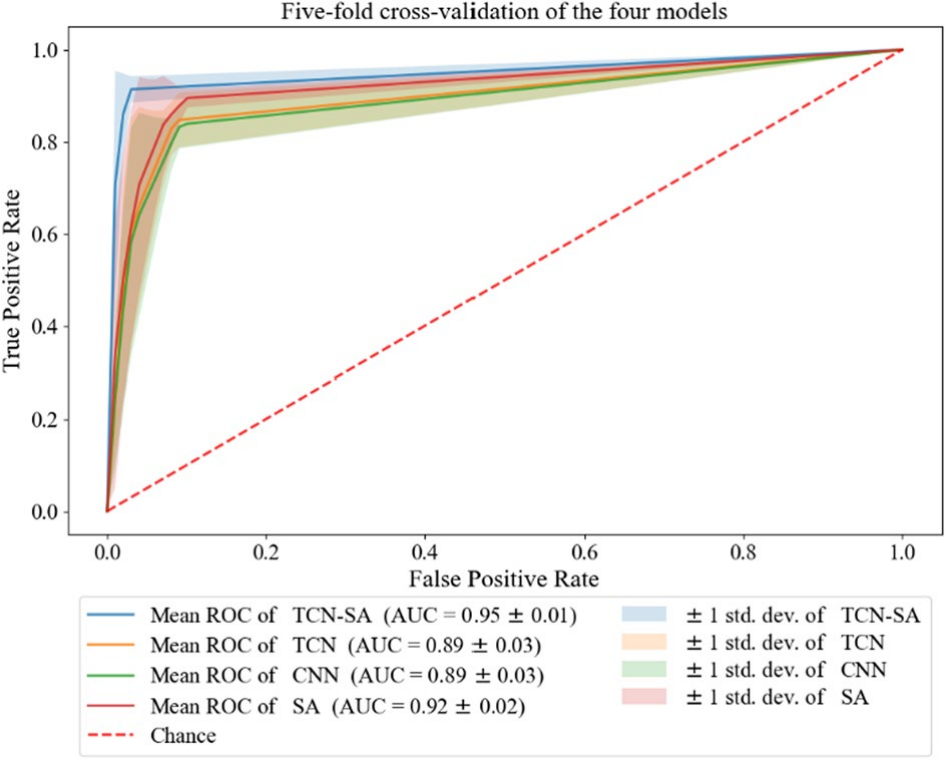
Average ROC curve, confidence interval, and average AUC of the four models in the fivefold cross-validation

At the performance testing stage, the four models were subject to fivefold cross-validation. The participants in each fold are listed in Table 1. The average accuracy, sensitivity, specificity, precision, and F1-score (with standard errors) of the four models are shown in Table 2. The average receiver-operating characteristic (ROC) curve and average area under the curve (AUC; with standard errors) are shown in Fig. 2. The accuracy, sensitivity, specificity, precision, and F1-score of the TCN-SA model were 95.50%, 91.22%, 98.72%, 98.20%, and 0.94699, respectively; these were the highest of all the models. Compared with the CNN and TCN models, our method showed average accuracy improvements of 5.86 and 5.26%, respectively, sensitivity improvements of 8.25 and 7.23%, respectively, specificity improvements of 3.96 and 3.69%, respectively, precision improvements of 5.52 and 5.00%, respectively, and F1-score improvements of 0.0719 and 0.0639, respectively. Compared with the CNN model, the TCN model showed improvements in the average accuracy, sensitivity, specificity, precision, and F1-score, which were 0.60%, 1.03%, 0.27%, 0.52%, and 0.0080, respectively.

**Table 1:** Participants in each fold

Fold	Participant IDs	
	From healthy control group	From children with EP
1	20,24,28,33	3,6
2	14,15,22,29,31	0,9,11
3	17,19,25,32	5 8,12
4	16,23, 27,30	1,2,13
5	18,21,26,34	4,7,10

**Table 2:** Performance of TCN-SA, TCN, SA, and CNN models

Methods	Accuracy (%)	Sensitivity (%)	Specificity (%)	Precision (%)	*F*_*1*_-scores (%)
TCN-SA	95.50 ± 1.69	91.22 ± 2.92	98.72 ± 0.93	98.19 ± 1.13	94.54 ± 1.49
TCN	90.23 ± 3.85	83.99 ± 6.38	95.04 ± 2.94	93.19 ± 2.58	88.15 ± 3.11
SA	92.50 ± 2.37	88.87 ± 2.17	95.08 ± 3.27	93.45 ± 3.14	91.06 ± 1.66
CNN	89.63 ± 3.57	82.96 ± 5.71	94.77 ± 3.27	92.67 ± 3.24	87.35 ± 2.61

Figure 2 illustrates that the ROC curve of the TCN-SA model is proximate to the optimal position in the upper left corner and possesses the most constrained confidence interval among the models studied. This signifies that the model conferred the most favorable critical value. Utilizing this point for classification yielded high sensitivity and specificity while ensuring the combined false-positive and false-negative rates remained low. The AUC area of the TCN-SA model was 0.95 ± 0.01, outperforming the other models by showcasing the highest mean and least variability. The AUC of the TCN, SA, and CNN models was 0.89 ± 0.03, 0.92 ± 0.02, and 0.89 ± 0.03, respectively.

In a comprehensive assessment using the F1-score to rank the fivefold cross-validation outcomes, the TCN-SA model maintains superiority in both training and testing accuracy and loss, eclipsing the best performances of three models (TCN, SA, and CNN) (See Appendix: Fig. 11).

**Fig. 3:**
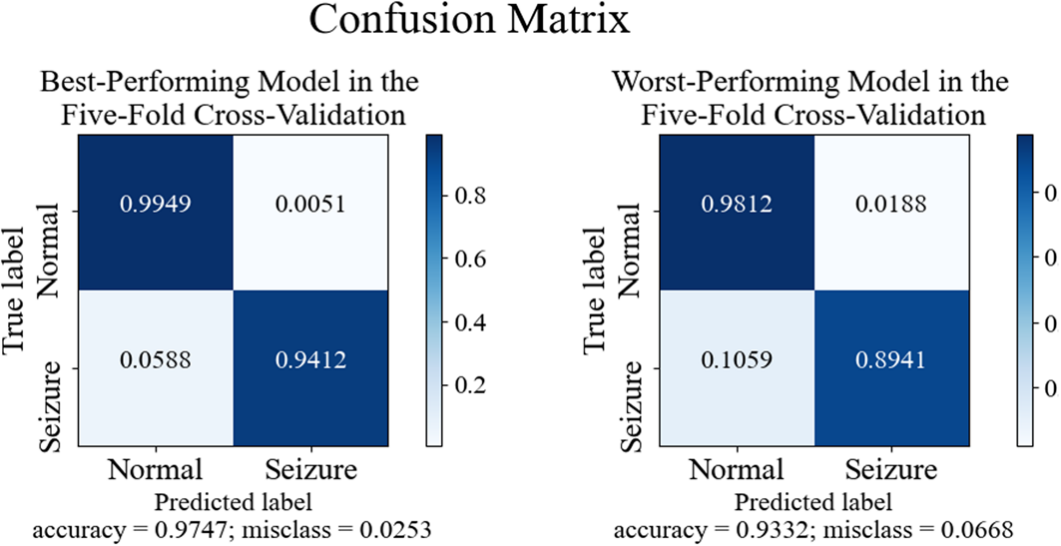
The best and worst confusion matrices for the fivefold cross-validations of the model

To further illustrate the performance of our model, we constructed a confusion matrix (Fig. 3). The color depth in the confusion matrix reflects accuracy and the values are marked in white within each color block. The matrix reveals specificity and sensitivity in the top left and lower right squares, while the upper right and lower left squares represent false-positive and false-negative rates, respectively. The sensitivity and specificity of the TCN-SA model with the best performance were 94.12% and 99.49%, respectively, and those of the worst performances were 89.41% and 98.12%, respectively.

**Fig. 4:**
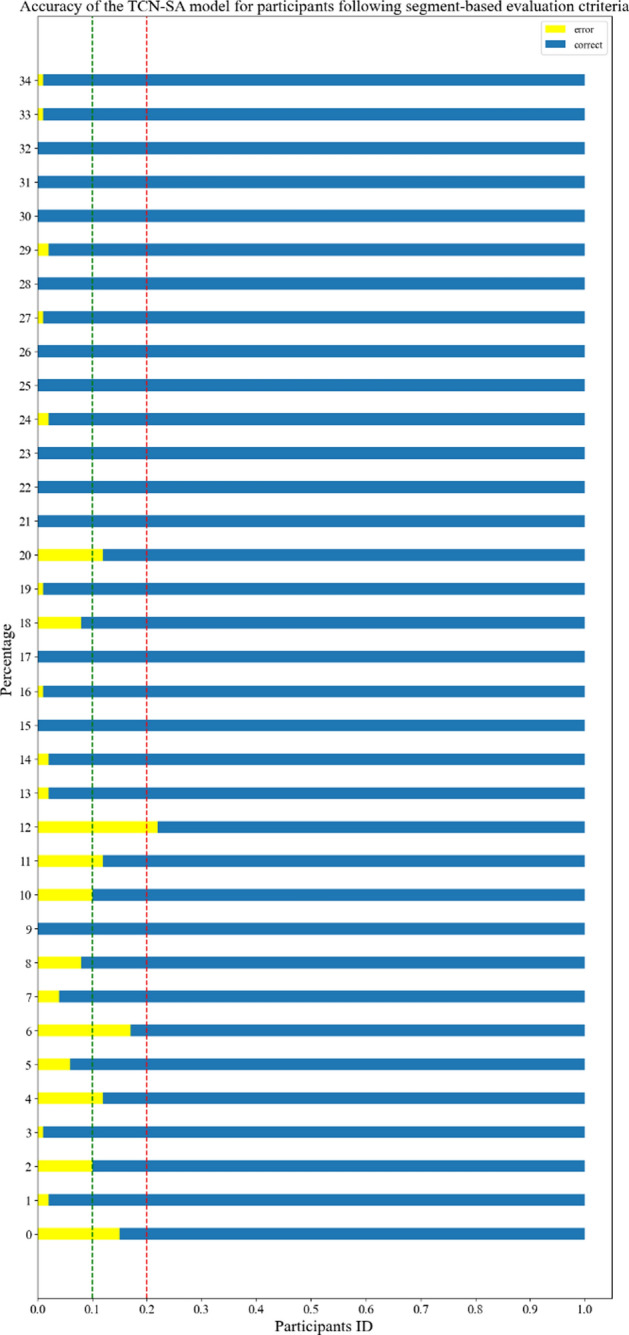
Accuracy of the TCN-SA model for participants following segment-based evaluation criteria

We further compared the overall accuracy of each participant in terms of the segment-based evaluation criteria across the four models. After obtaining the predictions for every segment for one participant, we concluded that the overall accuracy indicated the correct percentage of all the segments. In Fig. 4, the vertical axis labels the participants, with yellow and blue bar graphs representing the accurate and erroneous segment percentages, respectively, aided by red and green lines denoting thresholds at 0.2 and 0.1. The same notations hold for the other models (See Appendices Fig. 15).

In Fig. 4, the length of the yellow bar is < 0.2 (red line) for the majority of participants, indicating the overall accuracy exceeding 80% for almost all of the participants; of these, five participants had a yellow bar length greater than > 0.1 (green line), indicating that the overall accuracy surpasses 90% for the remaining 29 participants, with 12 participants even having accuracies reaching 100%. Some participants display overall accuracies below 80% (as seen in Appendices 15), with a few approximating a 50% overall accuracy.

### The bonn EEG dataset

**Fig. 5:**
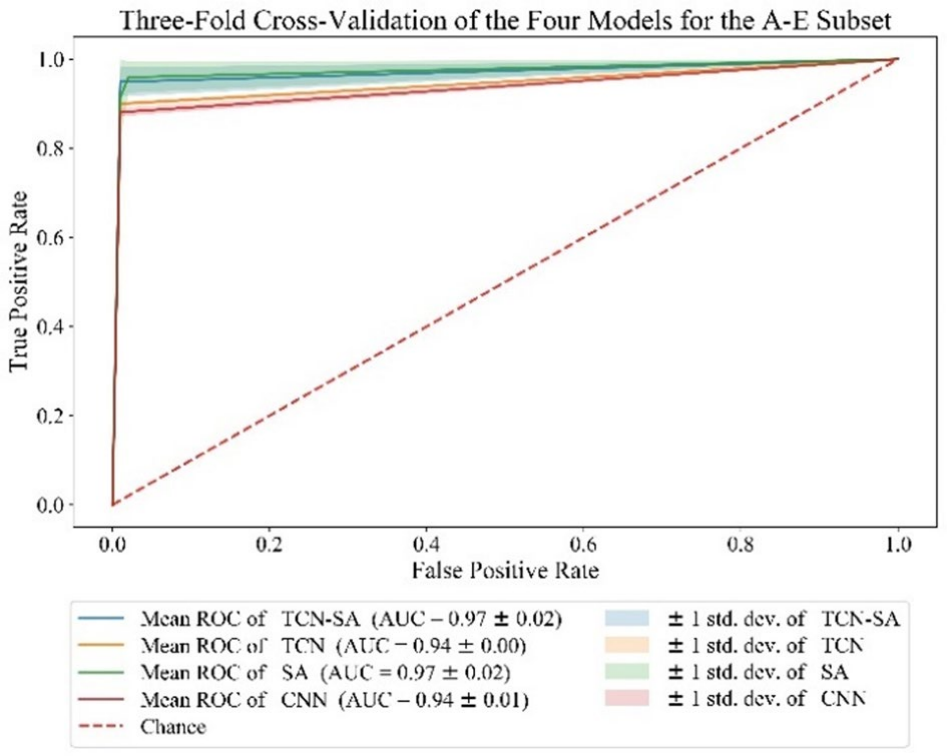
Average ROC curve, confidence interval, and average AUC of the four models in the threefold cross-validation for A-E

**Fig. 6:**
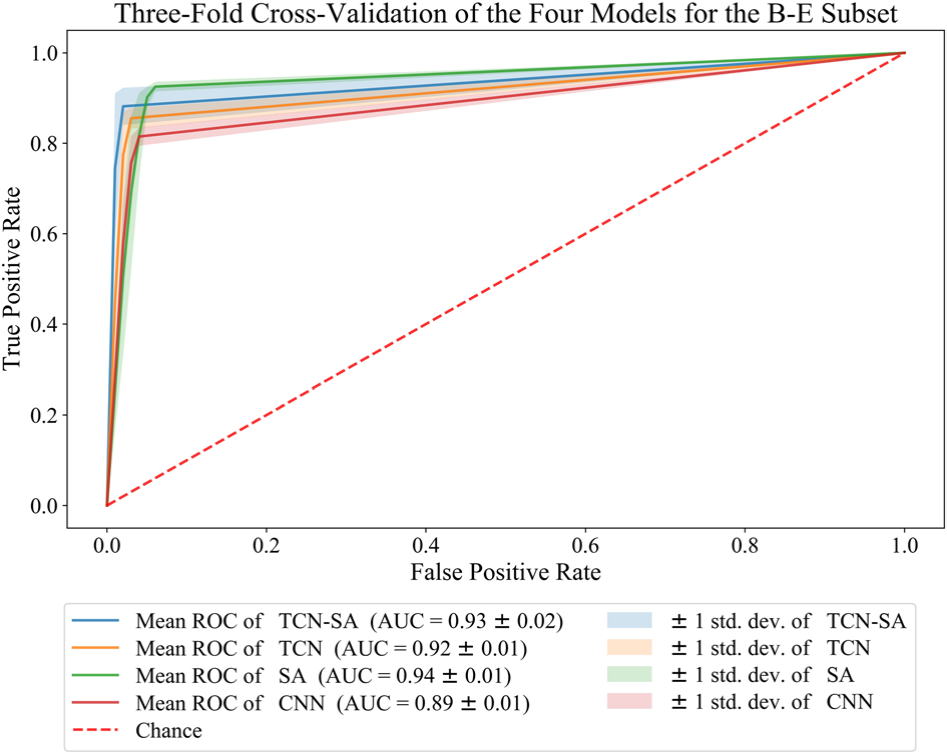
Average ROC curve, confidence interval, and average AUC of the four models in the threefold cross-validation for B-E

We conducted two experiments for the classification of healthy children and those with EP by combining different subsets, A-E and B-E, and adopting threefold cross-validation of the four models. The average accuracy, sensitivity, specificity, accuracy, and F1-score (with standard errors) of the four models are shown in Table 3. The average ROC curve and AUC are shown in Figs. 5 and 6. The accuracy, sensitivity, specificity, precision, and F1-score of the TCN-SA model for A-E were 97.37%, 94.88%, 99.91%, 99.91%, and 0.9730, respectively, whereas those for B-E were 93.50%, 88.07%, 99.00%, 98.86%, and 0.9311, respectively. For A-E, compared with the CNN and TCN models, our method showed average accuracy improvements of 3.57% and 2.48%, respectively, sensitivity improvements of 6.87% and 5.00%, respectively, and F1-score improvements of 0.0387 and 0.0267, respectively. For B-E, our method showed average accuracy improvements of 4.37% and 1.85%, respectively, sensitivity improvements of 6.81% and 2.69%, respectively, and F1-score improvements of 0.0491 and 0.0203, respectively, over the CNN and TCN models. Even if the performances of the SA and TCN-SA models were roughly the same in two experiments with the Bonn dataset [51], with the SA model even performing slightly better than the TCN-SA model, the performance of the SA model was the worst of the four models in experiments with our EEG data.

**Table 3:** Performances of the TCN-SA, TCN, SA, and CNN models with the Bonn dataset

Methods	Accuracy (%)	Sensitivity (%)	Specificity (%)	Precision (%)	*F*_*1*_-scores (%)
A-E					
TCN-SA	97.37 ± 1.74	94.88 ± 3.33	99.91 ± 0.06	99.91 ± 0.07	97.30 ± 1.79
TCN	94.89 ± 0.36	89.88 ± 0.51	99.91 ± 0.06	99.90 ± 0.07	94.63 ± 0.30
SA	97.54 ± 1.92	95.90 ± 3.55	99.17 ± 0.27	99.13 ± 0.31	97.46 ± 1.99
CNN	93.80 ± 0.60	88.01 ± 0.91	99.61 ± 0.11	99.56 ± 0.12	93.43 ± 0.56
B-E					
TCN-SA	93. 50 ± 2.37	88.07 ± 4.10	99.00 ± 0.56	98.86 ± 0.64	93.11 ± 2.54
TCN	91.65 ± 1.03	85.38 ± 2.38	97.97 ± 0.54	97.67 ± 0.65	91.08 ± 1.15
SA	94.11 ± 0.64	92.37 ± 1.13	95.88 ± 1.22	95.73 ± 1.31	94.00 ± 0.60
CNN	89.13 ± 0.94	81.26 ± 2.16	97.06 ± 0.60	96.50 ± 0.75	88.20 ± 0.95

Figures 5 and 6 show that the AUC for A-E with the TCN-SA, TCN, SA, and CNN models is 0.97 ± 0.02, 0.94 ± 0.00, 0.97 ± 0.02, and 0.94 ± 0.01, respectively, whereas that for B-E is 0.93 ± 0.02, 0.92 ± 0.01, 0.94 ± 0.01, and 0.89 ± 0.01, respectively. For A-E, the ROC curve of the TCN-SA model is closer to the upper left corner, with the TCN-SA and SA models having the largest AUC. For B-E, the ROC curve of the SA model is closer to the upper left corner, with the narrowest confidence interval of the curve.

In addition, the F1-score was used to sort the cross-validation models and compare the best performances of the four models with the worst performance of our model in the threefold cross-validation (See Appendix: Figs. 11, 12). The result shows that our model had the highest accuracy among the best performances of the four models. However, the worst performance of our model was at an intermediate level.

**Fig. 7:**
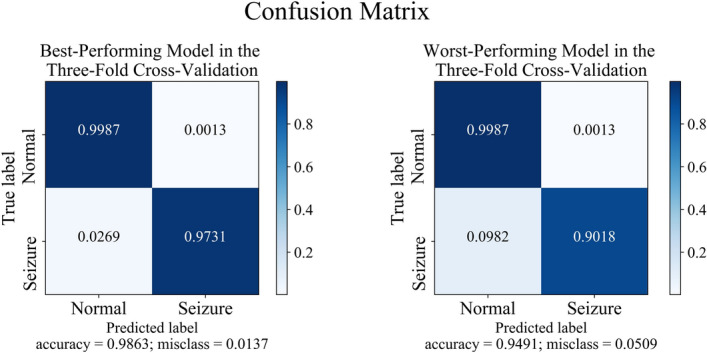
The best and worst confusion matrices for the threefold cross-validation of the TCN-SA model with the A-E subset

**Fig. 8:**
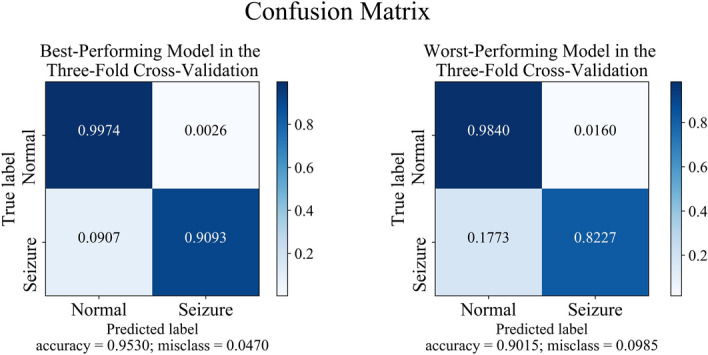
The best and worst confusion matrices for the threefold cross-validation of the TCN-SA model with the B-E subset

Finally, Figs. 7 and 8 show confusion matrices. In both experiments, the specificity of the TCN-SA model exceeded 98%. The sensitivity of the TCN-SA model at peak performance was 97.31% and 90.93%, respectively, for the A-E and B-E subsets. Its sensitivity at worst performance was 90.18% and 82.27%, respectively, for the A-E and B-E subsets.

## Discussion

In this paper, we propose a deep learning model (TCN-SA) for the automatic detection of EP in real world, which can adapt to complex conditions of the real-world scenario. By combining TCN with SA, our method provided a general model that can simultaneously handle the interictal and seizure EEGs; accordingly, our model is highly suitable for clinical applications. When only interictal EEG was included in EEG recordings, the TCN module extracted effective features with the same weighting from abnormal activity and normal activity in EEG sequences, and the SA module focused on the characteristic information of abnormal activity and increased the learned weight of abnormal activity to identify patients with EP during training. When interictal EEG and seizure EEG simultaneously in the EEG recordings, the TCN module extracted effective features with the same weighting from abnormal activity and normal activity in EEG sequences, and the SA module focuses on the abnormal activity of interictal EEG and seizure, changing a learned weight of interictal abnormal activity and seizure with their intensity difference. It can achieve efficient identification of patients with EP if the model prefers to learn information of seizure when the EEG recordings contain interictal EEG and seizure EEG simultaneously. This method was evaluated using two experiments performed with the pediatric EP dataset from the Shenzhen People’s Hospital and the Bonn dataset [51]. The results showed that our method performed well at the participant’s level in the EP detection task. The results of two datasets showed that our model can adapt to complex real-world scenarios and used as a clinically useful model for automatic detection of EP.

For our EEG dataset, the TCN-SA model showed the highest accuracy, sensitivity, specificity, precision, and F1-score of all four tested models. The performance of the TCN model was also better than that of the CNN model. This indicated that adding the SA layer enhanced the ability of the model to identify patients by focusing on key information, improving its overall performance. The ROC curve of the TCN-SA model had the best performance and narrowest confidence interval, indicating that our model had the best performance and stability. Regarding the overall accuracy for each participant in terms of segment-based evaluation criteria, the overall accuracy with our model was > 80% for almost all of the participants; for the 29 remaining participants, the overall accuracy exceeded 90%, and 14 participants had accuracies that reached 100%. For some participants, the overall accuracy was about 50% when the other three models were used. Despite the negative influence of individual differences, our model had the best performance in detection, reflecting its high stability. Compared with the CNN model, the TCN model had better performance in terms of the overall accuracy of participants in the healthy control group; it was also superior in the EP detection task.

For the Bonn dataset [51], the performances of the SA and TCN-SA models were roughly the same in two experiments (A-E and B-E). However, there are some clear differences in EP status between the Bonn [51] and our EEG datasets. The E subset consisted of mainly epileptic seizures, whereas our EEG dataset contained the interictal and ictal states, with the interictal state being predominant. Upon comparing the data status of our EEG and the Bonn datasets, we found that the SA model performed better in identifying epileptic seizures. However, when comparing the best performances of all four models, our model had the highest accuracy; its worst performance was at an intermediate level. It also confirmed that compared the task of identifying EP using the period of interictal state, the general deep learning model can easier achieve good performance identifying epileptic seizures.

Although the results of the TCN-SA model with the Bonn and our EEG datasets were roughly the same, the degree of difficulty of experiments with our EEG dataset was higher than that with the Bonn dataset [51]. First, the Bonn dataset [51] consisted of single-channel signals, whereas our EEG dataset comprised multi-channel signals. Compared with single-channel signals, multi-channel signals are more complex and redundant and hence contain more useful information regarding epileptic seizures. Second, as the experiment with our EEG dataset was evaluated based on subjects; the problem of inflating extrapolation ability was avoided using EEG fragments from the same subject only in either the training or testing set. Third, the E subset in the Bonn dataset [51] comprised epileptic seizures, whereas our EEG dataset contained the interictal and ictal states, with the interictal state being predominant; specifically, three subjects exhibited no seizures during the EEG recordings. Our model showed high classification accuracy in the experiment with the Bonn dataset [51], verifying that it could handle the task of automatically detecting EP from a general epileptic EEG dataset.

To further evaluate the effectiveness of our model, we compared it with other works for the automatic detection of EP from EEG signals. As shown in Table 4, the results of our method and those of other methods were evaluated using the Bonn dataset [51]. Our method appeared to perform equivalently to others. For the A-E subset, our method was second best but differed from the best method by only 0.63%. For the B-E subset, our method was the best.

**Table 4:** Comparing the performance of the TCN-SA model with that of other models

Method	Study	Accuracy (%)
A-E		
TCN-SA	Our model	**97.37**
STFT spectrogram + Effi-cientNet B7	Ilias et al. [62]	96.50
MWT + ApEn + ANN	Guo et al. [63]	96.00
Nonlinear prepossessing filter + LAMSTAR	Nigam et al. [64]	97.20
dWT + ME	Subasi [65]	94.50
1D-TP + ANN	Kaya et al. [66]	98.00
P-1D-CNN	Ihsan Ullah et al. [68]	99.90
LSTM	Ahmedt-Aristizabal et al. [67]	97.00
B-E		
TCN-SA	Our model	**93.50**
1D-TP + ANN	Kaya et al. [66]	93.00
LSTM	Ahmedt-Aristizabal et al. [67]	92.50
P-1D-CNN	Ihsan Ullah et al. [68]	99.00

Although our model showed high classification accuracy, it has some limitations. First, we only verified our model using a dataset from children with EP. Future research will aim to acquire adult EP patient data to broaden the model’s applicability. Moreover, although our model achieved great classification for the EEG dataset we collected, it cannot be utilized to locate seizures for online detection. In our EEG dataset, abnormal discharge segments and normal segments from the raw EEGs were extracted by us under the guidance of professional neurologists before data preprocessing. In the future work, the TCN-SA model can be utilized to locate seizures for online detection after the automatic data preprocessing so as to apply to the pre-consultation to the neurologist at the Outpatient Department [69–71]. Finally, our model also lacks interpretability. Although this is an effect of the general black-box nature of deep learning approaches, it is necessary to interpret models in the medical field [72]. We aim to improve the interpretability of our model by taking the advantage of outstanding machine learning algorithm in the automatic detection of epilepsy [73–75].

## Conclusions

In this study, the TCN-SA model was used for the first time for the automatic detection of EP from EEG data. The TCN extracts EEG features and the SA layer enhances the identification of key features, thereby lowering the computational cost and time. The TCN-SA model achieved 95.40% accuracy in the classification of EP among children; compared with the TCN, SA, and CNN models, its accuracy was improved by 5.33%, 6.79%, and 6.24%, respectively. In addition, our method achieved high classification accuracies with the Bonn dataset [51] (A-E and B-E subsets). The validity of the TCN-SA model shows that it is worthy of implementation for the automatic detection of EP from EEG data.

## Methods

### Data description

A new dataset that we generated ourselves was used to verify our model, and the Bonn dataset [51] was used as the external validation.

#### Our EEG data

We obtained EEG data from the Department of Pediatrics, Shenzhen People’s Hospital, China, between January 2019 and June 2021. The raw data were anonymized before analysis. This study was approved by the Ethics Committee of the School of Public Health, Sun Yat-sen University (No.2021–081), and informed consent was obtained from the research participants. In accordance with the international 10–20 system, an EEG instrument has 19 electrodes (FP1, FP2, F3, F4, C3, C4, P3, P4, O1, O2, F7, F8, T3, T4, T5, T6, Fz, Cz, and Pz) and two reference electrodes (A1 and A2). Resting-state EEGs were recorded at 500 Hz with a Nicolet recording system (Thermo Nicolet Corporation, USA). Based on the guidelines listed by the International Alliance Against Epilepsy [54], the inclusion and exclusion criteria were set as follows:

1. Inclusion criteria:

1. At least two unprovoked (or reflex) seizures occurring > 24 h apart
2. One unprovoked (or reflex) seizure and probability of further seizures similar to the general recurrence risk (at least 60%) after two unprovoked seizures
3. Diagnosis of an EP syndrome.

2. Exclusion criteria:

1. Other neurological diseases in addition to EP
2. Significant progressive disorders or unstable medical conditions requiring acute intervention
3. Cognitive impairments precluding psychiatric and clinical evaluations
4. Any history of anti-seizure medication use.

The EEG data from healthy children were normal, whereas those from children with EP covered their interictal and ictal states. In our dataset, the abnormal discharge segments were of two types: seizures in their ictal state, and spike and wave complexes in their interictal and ictal states. However, seizures were not recorded in the EEG data of every child with EP; three children did not have seizures when recording EEGs.

The dataset after selection consisted of 35 children is divided into two groups: one group of healthy children (*n* = 21, average age: 6.9 ± 3.6 years, male = 12, female = 9) and one group of children with EP (*n* = 14, average age: 7.6 ± 3.7, male = 8, female = 6). The two groups were homogeneous in terms of sex and age; the Chi-square test showed no significant differences in sex ($X^{2} \, < \,0.001$, $P=1.000$), and two-tailed Mann–Whitney *U* tests showed no significant differences in average age groups ($U=125.500$, $P=0.466$).

#### The bonn dataset

The Bonn dataset [51] has five subsets (A, B, C, D and E). Each contains 100 single-channel segments, and each signal lasts 23.6 s and was obtained at a sampling rate of 173.61 Hz. The EEG segments of subsets A and B were collected from five healthy volunteers, whereas those of subsets C, D and E were collected from five patients with EP. Table 5 shows details of the Bonn dataset [51]. All the segments were selected and removed from the continuous multi-channel EEG recordings after visual inspection for artifacts such as muscle activity and eye movements.

**Table 5:** Characteristics of the Bonn dataset

Subset	Subject	State	Electrode placement
A	Healthy	Awake state with eyes open	International 10–20 system
B	Healthy	Awake state with eyes closed	International 10–20 system
C	Epileptic	Interictal	Hippocampus opposite to hemisphere
D	Epileptic	Interictal	Epileptogenic zone
E	Epileptic	Ictal	Epileptogenic zone

### Data preprocessing

Under the guidance of professional neurologists, we extracted abnormal discharge segments from the raw EEGs of the children with EP and then extracted normal segments from the raw EEGs of the healthy children, without interference. Moreover, the raw data needed to be preprocessed before conducting formal EEG analysis. To reduce the computational burden, we downsampled the data at 100 Hz. First, high-pass filtering was carried out at a frequency of 1.6 Hz, after which low-pass filtering was carried out at 70 Hz. Then, band-pass filtering was used to remove the power frequency interference (50 Hz). Finally, the original data were divided into non-overlapping fragments with lengths of 2 s using a sliding time window. The preprocessed fragment set of each patient was thus obtained. Each fragment size was 19 (channels) × 200 (sampling points).

For the Bonn dataset [51], we also down sampled the data at 100 Hz and conducted the same preprocessing with filtration. The EEG signals were divided into non-overlapping fragments of equal size, with each fragment size being 1 (channels) × 100 (sampling points).

The data were standardized to the range of [0,1] using the following formula to prevent numerical overflows and improve prediction accuracy. These preprocessing steps were performed using the EEGLab toolbox [55] in MATLAB (MathWorks). Subsequently, standardized segmented data are then input into a neural network, correspond to an input size of 19(channels) × 200(sampling points), 1(channel) × 100(sampling points), respectively

1 $${{\text{Z}}}_{{{\text{x}}}_{{\text{it}}}}=\frac{{{\text{x}}}_{{\text{it}}}-{{\text{x}}}_{{\text{tmin}}}}{{{\text{x}}}_{{\text{tmax}}}-{{\text{x}}}_{{\text{tmin}}}}$$

### Model architecture

**Fig. 9:**
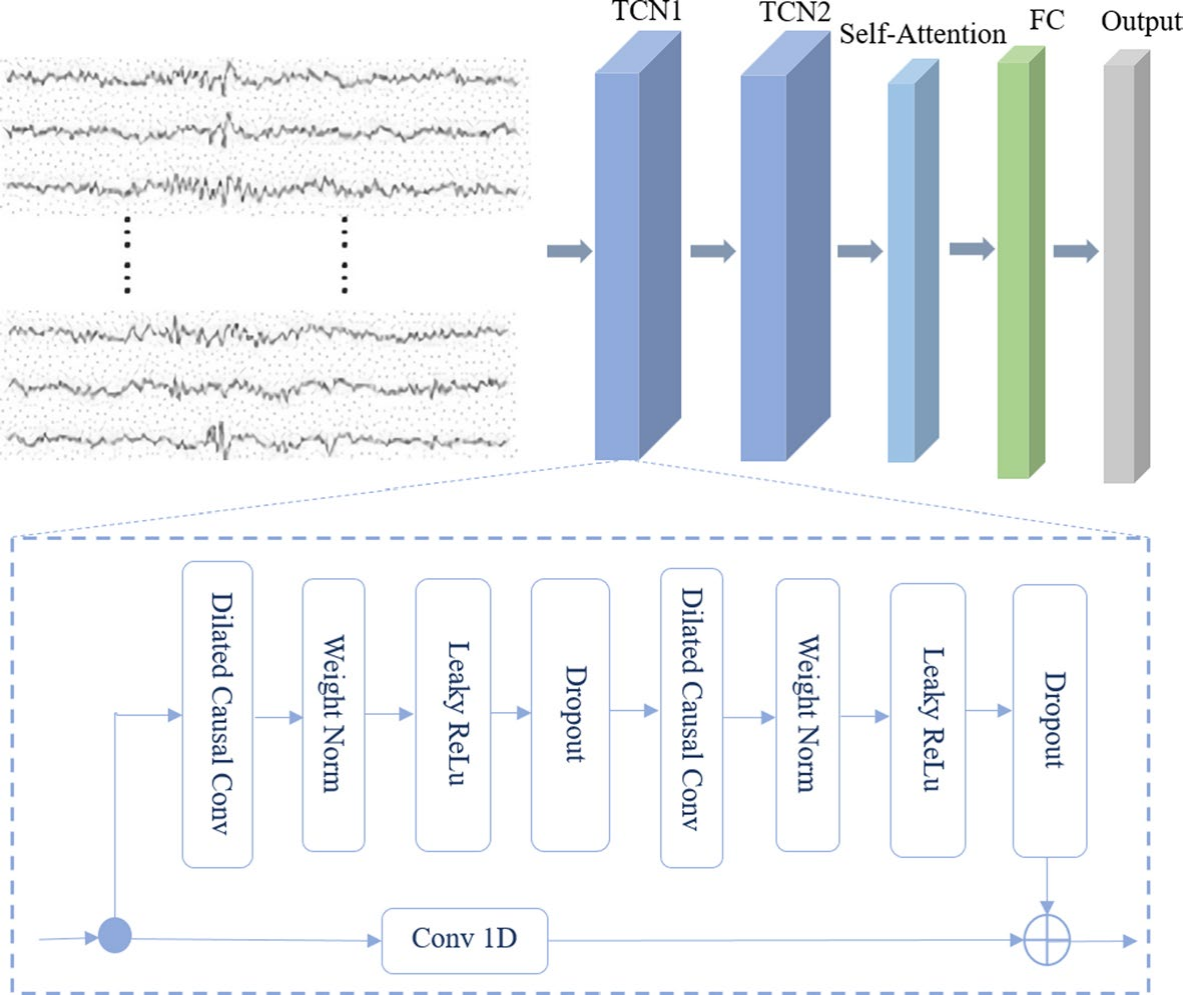
Overview of the proposed TCN-SA model

The architecture of the TCN-SA model primarily consists of the TCN and SA blocks, as shown in Fig. 9. In the model, the TCN block is utilized to learn sequences of EEGs in each sample after preprocessing, capture the long-term features of EEG signals, and then output feature sequences. The SA layer, placed after the TCN block, is used to obtain the inner links of feature sequences and compute associations between pairs of features to discriminate interictal and seizure EEGs. We used attention weights to increase the effectiveness of neural network training and obtain classification prediction outputs through the full connection.

The details of the proposed model are described in the following subsection.

#### Temporal convolutional neural network

Temporal convolutional neural network is a new type of CNN originally proposed by Lea et al. [41, 42]. This type of network is used to analyze input data by combining causal and dilated convolution. They adopt a residual network to generate the outcome.

##### Causal and dilated convolution

A key characteristic of a sequence model is that the prediction of each moment only depends on the observation of its historical moment and not on future observations. Causal convolution [41, 42] requires that the output of the current moment to be obtained only via a convolutional calculation using features of the historical moment. This implies that causal convolution is a one-way convolution from the historical moment to the current moment.

Causal convolution has an important advantage of supporting parallel operation but requires an infinite number of convolutions when it is adopted for super-long sequences. To overcome this, a dilated convolution was applied to causal convolution to dynamically change the receptive field size of causal convolution by adjusting the dilated value to reduce the number of convolutions. For any causal convolution layer, as the dilated value can increase in the form ${2}^{i} (i=\mathrm{number of convolution layer})$ when there is more than one layer of causal convolution in the network, the length of the historical sequence based on dilated convolution is determined by the following formula:

2 $$length=d \times \left(k-1\right)$$

where *d* represents the dilation factor, *k* represents the filter size and $length$ is the length of the historical sequence calculated.

**Fig. 10:**
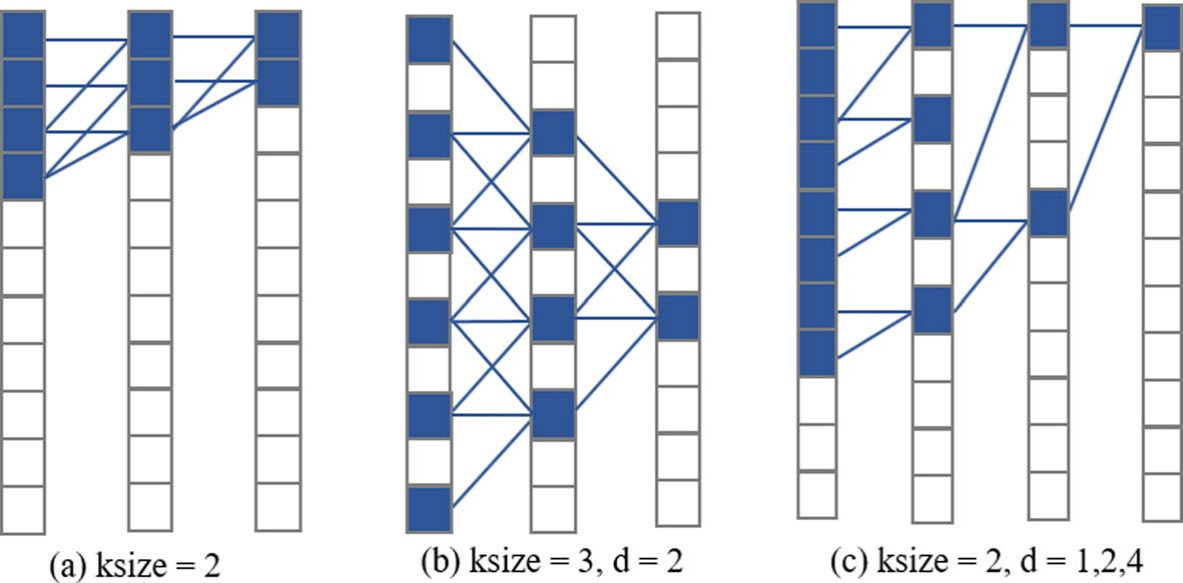
Causal and dilated convolution: **a** represents the causal convolution with a convolution kernel size of 2, **b** represents the dilated convolution with a convolution kernel size of 3, and (**c**) represents the causal convolution with a dilated value of 3 and convolution kernel size of 2

**Fig. 11:**
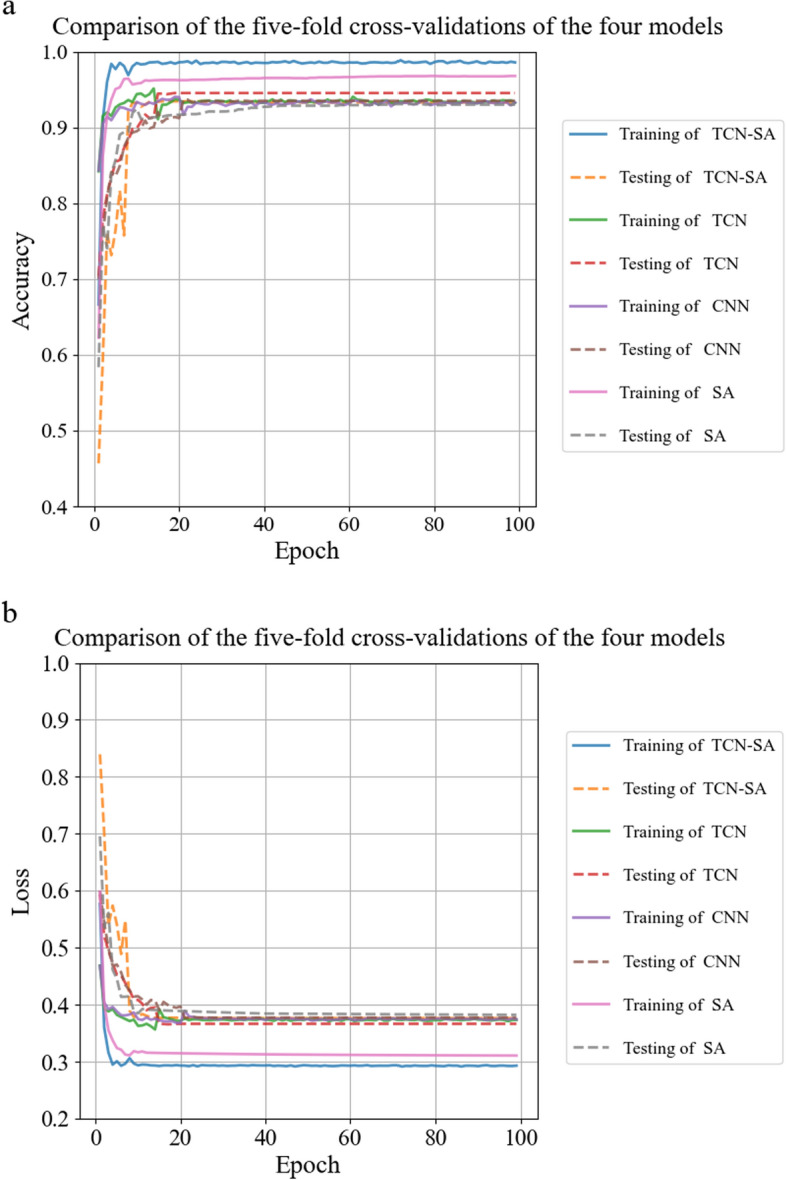
Accuracy and loss of the best and worst effects of the four models in the fivefold cross-validation. **a** Accuracy; **b** loss

**Fig. 12:**
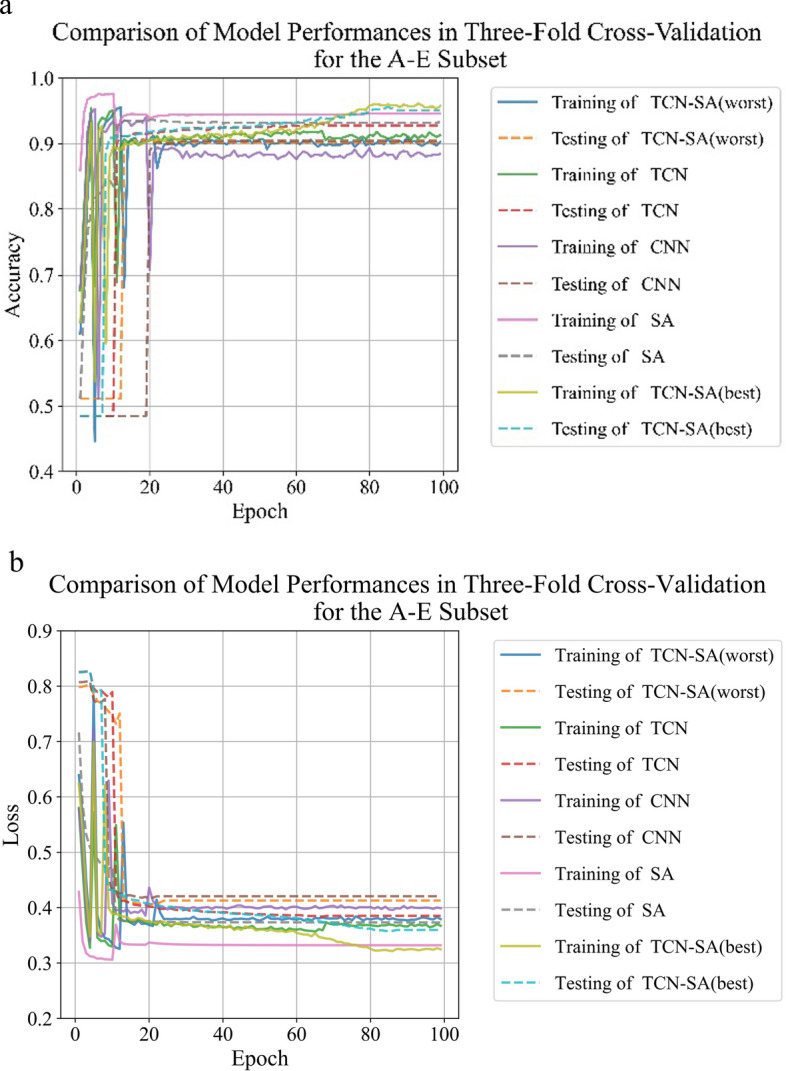
Accuracy and losses of the best and worst performances of the four models in the threefold cross-validation for A-E. **a** Accuracy, **b** loss

**Fig. 15:**
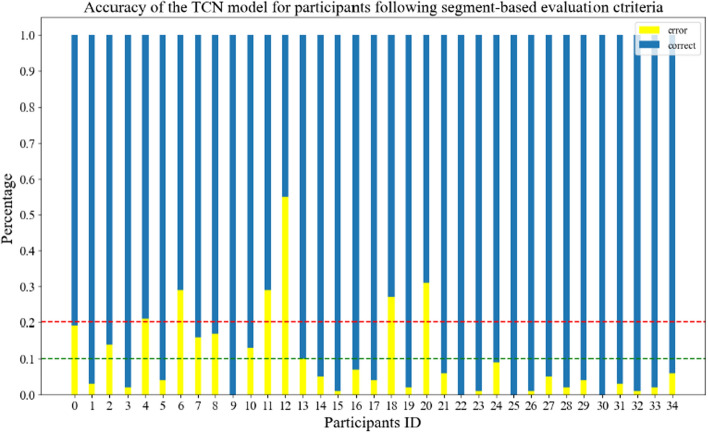
Accuracy of the TCN model for participants following segment-based evaluation criteria

Figure 10 shows an example of causal convolution combined with dilated convolution.

##### Residual module

For a residual module, the input is computed by combining causal dilated convolution with non-linear mapping, whereas the output is exported following a full connection layer. As each residual module contains both a dropout and weight normalization layer, the full connection layer not only improves the stability of the neural network but also ensures that the lengths of the input and output remain consistent. The formula is as follows:

3 $$o=activation\left(x+F\left(x\right)\right),$$

where $o$ and $x$ are the output and input to the model, respectively, and $activation$ represents the activation function.

#### SA mechanism

Attention mechanisms tend to focus on the attention of human beings, the underlying process of which is similar to that of vision. An attention mechanism can improve the performance of a model in a stepwise manner [56] by focusing on key information. There are four types of attention mechanism: softness of attention, forms of input features, input representations, and output representations [57]. The SA mechanism belongs to the category of input representations. In time-series models, the SA mechanism may weigh observations for each moment with the correlations between them. For multiple convolutional layers, the SA mechanism significantly compresses the characteristic matrix of the convolution output and retains important information. In addition, compared with the traditional sequence model that performs well in identifying long-term associations, SA has been more widely applied in various fields [58–61].

To measure self-attention, a data sequence of length of N was first encoded into $\mathrm{key }M=\left\{{m}_{1},{m}_{2},\dots ,{m}_{n-1},{m}_{n}\right\}$ and expressed as a key value in the form $\left(M,V\right)=[\left({m}_{1},{v}_{1}\right), \left({m}_{2},{v}_{2}\right), \dots .,({m}_{n-1},{v}_{n-1}),({m}_{n},{v}_{n})]$. Note that ${m}_{i}$ corresponds uniquely to one in *V*. *M* and *V* are different representations of a data sequence that indicate attention distribution and contextual information [55], respectively. For each query $Q$ from *M*, similarity with all values of $M$ were calculated using a score function known as the scaled multiplicate [34]; the standardized attention score of each key $M$ to $V$ was then obtained using the function $softmax$. Finally, sequence $V$ was weighted to the normalized attention score as the attention weight and named the SA value. The formula is as follows:

4 $$attention\left(Q,M,V\right)=softmax\left(\frac{Q{M}^{T}}{\sqrt[2]{{d}_{m}}}\right)V,$$

where $d$ represents the dimensions of an input sequence.

### Training and testing

At the model comparison stage, we randomly selected 70% of the subjects from whom we obtained EEG data to be part of the training set, the remaining subjects were made part of the testing set, and we randomly selected 20% of the training set to serve as a validation set. The training and testing sets comprised the fragment set of the corresponding subjects. To avoid inflating the extrapolation ability, we ensured that there were no fragments from the same subject simultaneously in the training and testing sets. At the performance testing stage, we utilized fivefold cross-validation to evaluate the stability and reliability of the TCN-SA model for the task of automatically detecting EP. For this cross-validation technique, the training set was split into five groups, with each group containing 6–8 participants. This process was repeated five times, with one group in each run serving as a source of validation data and the rest of the groups serving as the training set. In other words, we randomly selected 20% of the subjects from each training set to serve as a validation set. The validation sets were then used to adjust the hyper-parameters during model training to avoid overfitting.

To compare with our previous study, we verified our model using the Bonn dataset [51] and threefold cross-validation. The EEG data were divided into three subsets, of which two served as the training sets and one served as the testing set. This process was repeated 3 times, and the average value of the evaluation measurement over these three runs was computed. Each validation set was selected from 20% of each training set.

### Experiment settings

The experiment was performed on the high-performance computing cluster platform at the School of Public Health, Sun Yat-sen University. We also used Python 3.8, run on an Intel Xeon E5-2682 v4 CPU, a GTX1080TI GPU and a CUDA11.0 acceleration environment using the PyTorch deep learning framework. Table 6 shows our model parameters settings.

**Table 6:** Parameters settings the TCN-SA model

Parameters	Value
Kernel size	4
Learning rate	0.01
Epochs	100
Batch size	12
Optimization function	‘SGD’
Output parameters	2 neurons, Softmax

In our work, the computational complexity of our model measured by the number of floating-point operations (FLOPs) and model parameters. The experimental results show that our model parameters was 0.42 M, and the FLOPs was 4.04 GFLOPs.

Tables 7–9 present a comparative analysis of our model across various configurations of layers, kernel sizes, and activation functions. For layers and kernel size, we compared our model and TCN model when the SA block shares the layers and kernel size because of the entirety of the TCN and SA blocks. In Table 7, accuracy of TCN model increased with the number of layers, while accuracy of TCN-SA model was stable (layers ≤ 3), and the same experimental results can be shown in Table 8 (kernel size ≤ 8), so layers with 2 and kernel size with 4 would be the optimal choice with high efficiency and low energy consumption. The different change between TCN model and TCN-SA model verified that the SA layer can effectively attribute for the output accuracy when it focuses on the abnormal activities of interictal EEG and seizure by changing the learning weights of the TCN during neural network training. For activation function, we compared three models. As shown in Table 9, the softmax activation function yielded high and stable output accuracy among three models (TCN, SA, TCN-SA), and loss function corresponding to softmax activation function is cross-entropy.

**Table 7:** Accuracy (%) of TCN-SA, TCN models with different layers

Methods	Layers		
	2	3	4
TCN-SA	96.61	96.92	59.05
TCN	88.88	90.14	91.02

**Table 8:** Accuracy (%) of TCN-SA and TCN models with different kernel size

Methods	Kernel size						
	2	4	6	8	16	32	64
TCN-SA	96.86	96.61	96.48	96.92	59.05	59.05	59.05
TCN	87.75	88.88	89.51	59.05	59.05	59.05	59.05

**Table 9:** Accuracy (%) of TCN-SA, TCN, and SA models with different activation function

Methods	Activation function		
	Sigmoid	Softmax	Tanh
TCN-SA	93.78	96.61	59.05
TCN	88.63	88.88	88.13
SA	40.95	90.14	58.98

### Evaluation criteria

An average preformation of cross-validation was used to obtain stable results for our network model. The performance of a network model is measured by five indicators: precision, sensitivity, specificity, F1-score, and accuracy.

## Figures and Tables

**Fig. 13:**
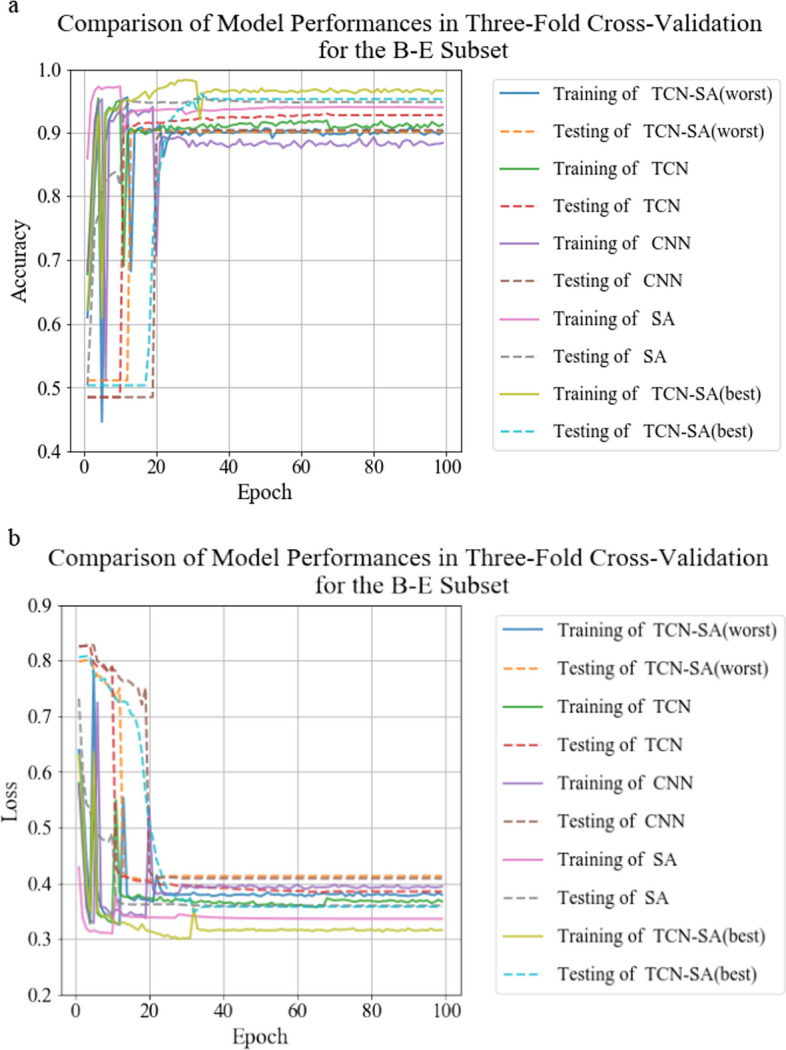
Accuracy and losses of the best and worst performances of the four models in the threefold cross-validation for B-E. **a** Accuracy, **b** loss

**Fig. 14:**
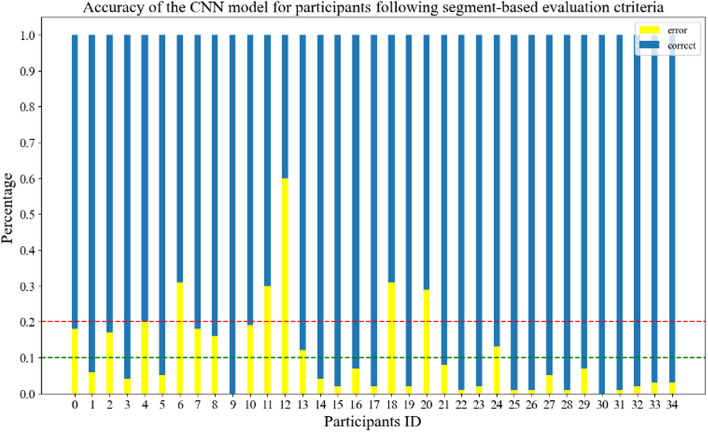
Accuracy of the CNN model for participants following segment-based evaluation criteria

**Fig. 16:**
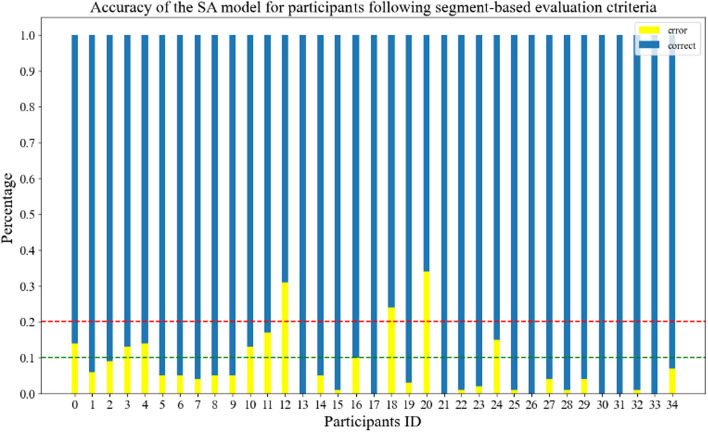
Accuracy of the SA model for participants following segment-based evaluation criteria

## Data Availability

The data presented in this study are available on request from the corresponding author. The data are not publicly available due to issues of participant confidentiality.

## Appendix

A. Comparison of the five-fold cross-validations of the four models.

B. Accuracy of the three models for participants following segment-based evaluation

See Figs. 11, 12, 13, 14, 15, 16

## Abbreviations

AUC: Area under curve
CNN: Convolutional neural network
EEG: Electroencephalogram
EP: Epilepsy
GRU: Gated recurrent unit
LSTM: Long short-term memory
RNN: Recurrent neural network
ROC: Receiver-operating characteristic
SA: Self-attention
TCN: Temporal convolutional neural network
